# Bis(diisopropyl­ammonium) hexa­chlorido­stannate(IV)

**DOI:** 10.1107/S1600536812043371

**Published:** 2012-10-24

**Authors:** Guido J. Reiss, Cora Helmbrecht

**Affiliations:** a Institut für Anorganische Chemie und Strukturchemie, Lehrstuhl II: Material- und Strukturforschung, Heinrich-Heine-Universität Düsseldorf, Universitätsstrasse 1, D-40225 Düsseldorf, Germany

## Abstract

The title compound, (C_6_H_16_N)_2_[SnCl_6_], crystallizes with one diisopropyl­ammonium cation lying on a general position and the hexa­chloridostannate(IV) anion about a centre of inversion. The [SnCl_6_]^2−^ anion undergoes a slight distortion from octa­hedral symmetry as the result of the formation of four unforked charge-supported N—H⋯Cl hydrogen bonds. The hydrogen bonds between the cations and anions form layers perpendicular to [101]. These layers are built by 24-membered rings which can be classified with an *R*_8_^8^(24) graph-set descriptor. According to this hydrogen-bonding motif, the title compound is isostructural with (C_6_H_16_N)_2_[IrCl_6_].

## Related literature

For related diisopropyl­ammonium salts, see: Fu *et al.* (2011 ▶); Reiss (1998 ▶, 2002 ▶, 2012 ▶); Reiss & Helmbrecht (2012 ▶); Reiss & Meyer (2011 ▶). For layered structures, see: Cameron *et al.* (1983 ▶); Holl & Thewalt (1986 ▶); Rademeyer *et al.* (2007 ▶). For potassium hexa­halogenidometalates, see: Abrahams *et al.* (1989 ▶); Amilius *et al.* (1969 ▶); Boysen & Hewat (1978 ▶); Coll *et al.* (1987 ▶); Hinz *et al.* (2000 ▶). For spectroscopy of hexa­chloridostannate(IV) salts, see: Brown *et al.* (1970 ▶); Ouasri *et al.* (2001 ▶). For graph-set theory and its applications, see: Etter *et al.* (1990 ▶); Grell *et al.* (2002 ▶).
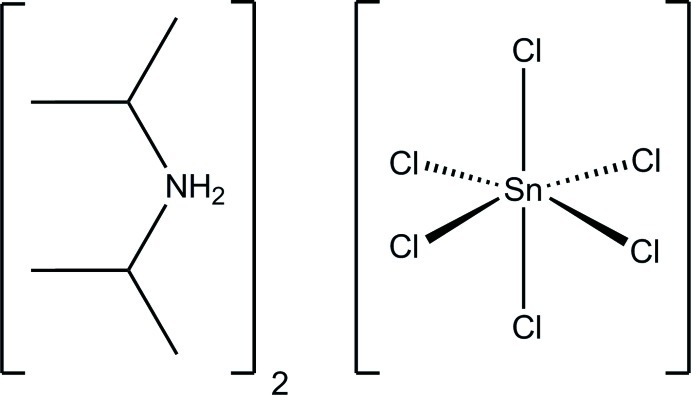


## Experimental

### 

#### Crystal data

(C_6_H_16_N)_2_[SnCl_6_]
*M* *_r_* = 535.81
Monoclinic, 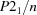

*a* = 9.54362 (13) Å
*b* = 11.98179 (19) Å
*c* = 9.90669 (14) Å
β = 92.9406 (14)°
*V* = 1131.33 (3) Å^3^
*Z* = 2
Mo *K*α radiation
μ = 1.83 mm^−1^
*T* = 100 K
0.33 × 0.27 × 0.08 mm

#### Data collection

Oxford Diffraction Xcalibur Eos diffractometer
Absorption correction: numerical (*CrysAlis PRO*; Oxford Diffraction, 2009 ▶) *T*_min_ = 0.634, *T*_max_ = 0.922
11414 measured reflections
4972 independent reflections
4468 reflections with *I* > 2σ(*I*)
*R*_int_ = 0.024

#### Refinement

*R*[*F* ^2^ > 2σ(*F* ^2^)] = 0.021
*wR*(*F* ^2^) = 0.048
*S* = 1.02
4972 reflections
120 parameters
H atoms treated by a mixture of independent and constrained refinement
Δρ_max_ = 0.53 e Å^−3^
Δρ_min_ = −0.57 e Å^−3^

### 

Data collection: *CrysAlis PRO* (Oxford Diffraction, 2009 ▶); cell refinement: *CrysAlis PRO*; data reduction: *CrysAlis PRO*; program(s) used to solve structure: *SHELXS97* (Sheldrick, 2008 ▶); program(s) used to refine structure: *SHELXL97* (Sheldrick, 2008 ▶); molecular graphics: *DIAMOND* (Brandenburg, 2012 ▶); software used to prepare material for publication: *publCIF* (Westrip, 2010 ▶).

## Supplementary Material

Crystal structure: contains datablock(s) I, global. DOI: 10.1107/S1600536812043371/mw2089sup1.cif

Structure factors: contains datablock(s) I. DOI: 10.1107/S1600536812043371/mw2089Isup2.hkl

Supplementary material file. DOI: 10.1107/S1600536812043371/mw2089Isup3.mol

Additional supplementary materials:  crystallographic information; 3D view; checkCIF report

## Figures and Tables

**Table 1:** Hydrogen-bond geometry (Å, °)

*D*—H⋯*A*	*D*—H	H⋯*A*	*D*⋯*A*	*D*—H⋯*A*
N1—H11⋯Cl1	0.881 (16)	2.541 (16)	3.3449 (10)	152.1 (13)
N1—H12⋯Cl2^i^	0.864 (15)	2.488 (15)	3.3507 (10)	176.0 (14)

Symmetry code: (i) 
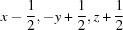
.

**Fig. 1.**
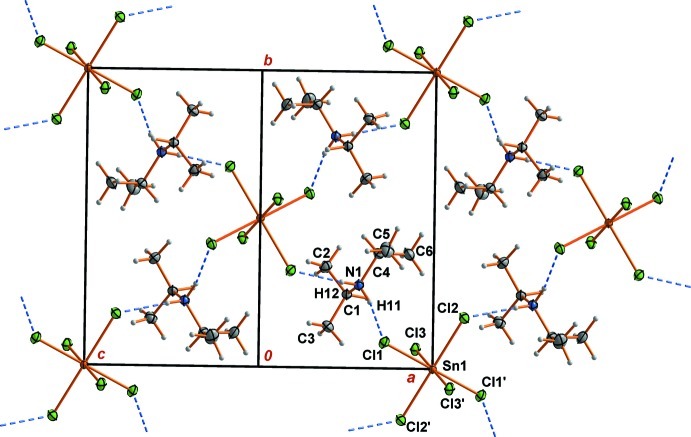
: View along [101] of the hydrogen bonded polymer layer of the title structure (Ellipsoids are drawn at the 60% probability level, ' = 2 - x, -y, -z).

**Fig. 2.**
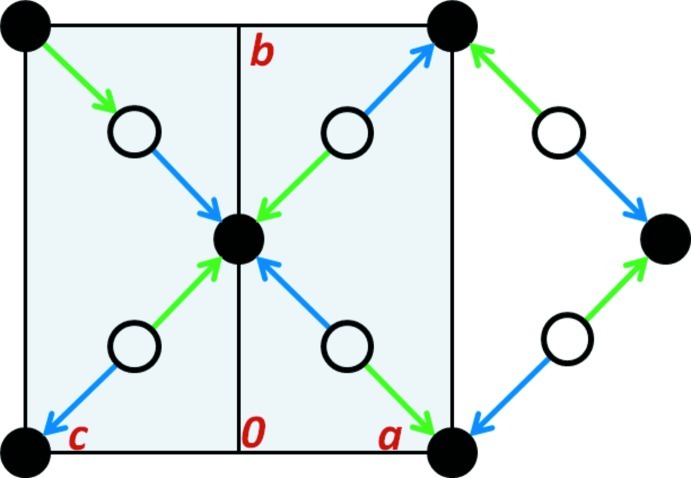
: Constructor graph (Grell et al., 2002) of that part of the title structure shown in Fig.1.

## supplementary crystallographic information

### Comment

Even though there are more than one hundred diisopropylammonium (*dipH*) salt structures listed in the Cambridge Crystallographic Data Base only a limited number of halogenidometalate-containing salts are reported: [SiF_6_]^2-^ (Reiss, 1998); [IrCl_6_]^2-^ (Reiss, 2002); [FeCl_4_]^-^ (Reiss, 2012), [CuCl_4_]^2-^ (Reiss & Helmbrecht, 2012). Recently the simple *dipH* chloride has attracted much attention as it is a ferroelectric solid with a high phase transition temperature (Fu *et al.*, 2011). This study on (*dipH*)_2_[SnCl_6_] is part of our long standing interest on the principles of arrangement of simple *dipH* salts (Reiss & Meyer, 2011).

The title compound (*dipH*)_2_[SnCl_6_] crystallizes with one *dipH* cation in a general position and one [SnCl_6_]^2-^ anion located on a center of inversion. The C–N and C–C bond lengths and the bond angles of the cation are in the expected range. The [SnCl_6_]^2-^ anion adopts a distorted octahedral geometry (angles between 89.00 (1) and 91.00 (1)°). The cations and anions are connected by medium-strong, charge-supported hydrogen bonds (Table 1) between the NH_2_^+^ groups and their neighbouring chlorine atoms (Fig. 1). Only four out of six chlorido ligands of each [SnCl_6_]^2-^ anion are involved with the Sn–Cl bonds participating in hydrogen bonding significantly longer (2.4359 (3) and 2.4527 (3) Å) than the two others (2.4055 (3) Å). This bonding situation results in the formation of two-dimensional layers in the [101] plane, whose characteristic motif is an annealed, 24-membered, wavy, hydrogen bonded ring (Fig. 1) with the graph-set descriptor *R*_8_^8^(24) (Etter *et al.*, 1990). This second level graph-set is shown in Fig. 2 as part of the constructor graph (Grell *et al.* 2002). The two other representative second level graph-sets are C_4_^4^(12) which run along [11–1] and C_2_^2^(6) which represents the bent connection of one [SnCl_6_]^2-^ anion with two *dipH* cations. The shortest H···Cl distance of the Cl3 is with 2.938 (16) Å roughly 0.5 Å longer than the two other H···Cl bonds. The acute N-H···Cl3 angle of 131.7 (12) ° supports our interpretation that the Cl3 atom is not involved in any significant hydrogen bond.

Analogous layered structures are also known for other (*R*_n_NH_4-n_)_2_[SnCl_6_] salts and have been discussed in detail (Holl & Thewalt, 1986; Cameron *et al.* 1983, Rademeyer *et al.* 2007).With the title compound featuring 24-membered hydrogen bonded rings, composed of four [SnCl_6_]^2-^ anions and four *dipH* ions, it is isostructural but not isotypical to (*dipH*)_2_[IrCl_6_] (Reiss, 2002). Whilst in (*dipH*)_2_[IrCl_6_] two crystallographically independent layers are present, in the title structure identical crystallographically dependent layers are stacked. The difference between the two structures is in the ring size of 11.9818 (2) / 14.1040 (2) Å (Fig. 1) for the latter and only 10.396 (1) / 13.638 (1) Å for the former and seems to be due to a more simple packing of the bulky isopropyl groups in the title structure. A structural relationship between the (*dipH*)_2_[IrCl_6_] and the K_3_[MoCl_6_] types of structures (Amilius *et al.*, 1969; Coll *et al.*, 1987; Hinz *et al.*, 2000) has been discussed (Reiss, 2002). In this structural family, the directly related higher symmetry K_2_[TeBr_6_] type (Abrahams *et al.*, 1989; Boysen & Hewat, 1978) exists which could be similarly compared to the title structure.

The Raman spectrum of (*dipH*)_2_[SnCl_6_] shows the Raman-active bands (ν_1_, ν_2_ and ν_5_) of the [SnCl_6_]^2-^ anions. Additionally a medium-strong band at 170 cm^-1^ is assigned to the ν_4_ mode which becomes Raman-active due to the distortion of the [SnCl_6_]^2-^ anion (Ouasri *et al.* 2001). Furthermore the band at 77 cm^-1^ represents a characteristic lattice mode for related compounds (Brown *et al.*, 1970).

### Experimental

(*dipH*)_2_[SnCl_6_] was prepared by dissolving 208 mg (2.05 mmol) diisopropylamine and 360 mg (1.02 mmol) tin(IV) chloride in 5 mL of concentrated hydrochloric acid (37 percent). In one to two days under ambient conditions colourless rhombic crystals were obtained by slow evaporation of the solvent. The Raman spectrum was measured using a *Bruker MULTIRAM* spectrometer (Nd:YAG-Laser at 1064 nm; RT-InGaAs-detector; resolution: 2 cm^-1^); 4000–70 cm^-1^: 3140(*w*), 3087(*w*), 2994(*s*), 2982(*m*, *sh*), 2970(*m*), 2948(*m*), 2908(*m*), 2700(*w*), 1574(*w*), 1479(*w*), 1458(*m*), 1411(*w*), 1342(*w*), 1296(*w*), 1196 (*w*, *sh*), 1184(*w*), 1168(*w*), 1142(*w*), 1084(*w*), 968(*w*), 957(w), 912(*w*), 880(*vw*), 824(*w*), 799(*m*), 468(*w*), 439(*w*), 309(*vs*; ν_1_, Sn–Cl), 235 (*m*, *br*; ν_2_, Sn–Cl), 170 (*s*; ν_4_, Sn–Cl), 159 (*s*; ν_5_, Sn–Cl), 77 (*m*; lattice mode). – IR spectroscopic data were recorded on a Digilab FT3400 spectrometer using a MIRacle ATR unit (Pike Technologies); 4000–560 cm^-1^: 3134(*vs*), 3082(*s*), 2991(*m*), 2981(*m*), 2969(*m*), 2945(*w*), 2835(*w*, *br*), 2712(*w*), 2442(*w*), 2391(*w*), 1620(*w*, *br*), 1573(*s*), 1472(*w*), 1466(*w*), 1458(*w*), 1425(*m*), 1395(*s*), 1384(*m*), 1358(*w*), 1342(*vw*), 1316(*w*), 1196(*w*), 1183(*m*), 1166(*w*), 1141(*m*), 1097(*m*), 969(*w*), 943(*w*), 877(*w*), 824(*w*), 798(*vw*).

### Refinement

All hydrogen atoms were identified in difference syntheses. The hydrogen atoms of the methyl groups were idealized and refined using rigid groups allowed to rotate about the C—C bond (AFIX 137 option of the *SHELXL97* programme). For each methyl group one common *U*_iso_ value was refined. The coordinates of hydrogen atoms belonging to the CH and NH_2_ groups were refined freely. The *U*_iso_(H) values of the two hydrogen atoms of the NH_2_ group were refined unrestricted.

### Crystal data

**Table:**

(C_6_H_16_N)_2_[SnCl_6_]	*F*(000) = 540
*M**_r_* = 535.81	*D*_x_ = 1.573 Mg m^−^^3^
Monoclinic, *P*2_1_/*n*	Mo *K*α radiation, λ = 0.71073 Å
Hall symbol: -P 2yn	Cell parameters from 8237 reflections
*a* = 9.54362 (13) Å	θ = 2.9–36.3°
*b* = 11.98179 (19) Å	µ = 1.83 mm^−^^1^
*c* = 9.90669 (14) Å	*T* = 100 K
β = 92.9406 (14)°	Plate, colourless
*V* = 1131.33 (3) Å^3^	0.33 × 0.27 × 0.08 mm
*Z* = 2	

### Data collection

**Table:**

Oxford Diffraction Xcalibur Eos diffractometer	4972 independent reflections
Radiation source: fine-focus sealed tube	4468 reflections with *I* > 2σ(*I*)
Graphite monochromator	*R*_int_ = 0.024
Detector resolution: 16.2711 pixels mm^-1^	θ_max_ = 35.0°, θ_min_ = 2.9°
ω scans	*h* = −15→15
Absorption correction: numerical (*CrysAlis PRO*; Oxford Diffraction, 2009)	*k* = −19→19
*T*_min_ = 0.634, *T*_max_ = 0.922	*l* = −12→15
11414 measured reflections	

### Refinement

**Table:**

Refinement on *F*^2^	Secondary atom site location: difference Fourier map
Least-squares matrix: full	Hydrogen site location: inferred from neighbouring sites
*R*[*F*^2^ > 2σ(*F*^2^)] = 0.021	H atoms treated by a mixture of independent and constrained refinement
*wR*(*F*^2^) = 0.048	*w* = 1/[σ^2^(*F*_o_^2^) + (0.0186*P*)^2^] where *P* = (*F*_o_^2^ + 2*F*_c_^2^)/3
*S* = 1.02	(Δ/σ)_max_ = 0.001
4972 reflections	Δρ_max_ = 0.53 e Å^−^^3^
120 parameters	Δρ_min_ = −0.57 e Å^−^^3^
0 restraints	Extinction correction: *SHELXL97* (Sheldrick, 2008), Fc^*^=kFc[1+0.001xFc^2^λ^3^/sin(2θ)]^-1/4^
Primary atom site location: structure-invariant direct methods	Extinction coefficient: 0.0041 (4)

### Special details

**Table:**

Experimental. Absorption correction: CrysAlisPro (Oxford Diffraction, 2009). Numerical absorption correction based on gaussian integration over a multifaceted crystal model.
Geometry. All esds (except the esd in the dihedral angle between two l.s. planes) are estimated using the full covariance matrix. The cell esds are taken into account individually in the estimation of esds in distances, angles and torsion angles; correlations between esds in cell parameters are only used when they are defined by crystal symmetry. An approximate (isotropic) treatment of cell esds is used for estimating esds involving l.s. planes.
Refinement. Refinement of *F*^2^ against ALL reflections. The weighted *R*-factor *wR* and goodness of fit *S* are based on *F*^2^, conventional *R*-factors *R* are based on *F*, with *F* set to zero for negative *F*^2^. The threshold expression of *F*^2^ > σ(*F*^2^) is used only for calculating *R*-factors(gt) *etc*. and is not relevant to the choice of reflections for refinement. *R*-factors based on *F*^2^ are statistically about twice as large as those based on *F*, and *R*- factors based on ALL data will be even larger.

### Fractional atomic coordinates and isotropic or equivalent isotropic displacement parameters (Å^2^)

**Table:**

	*x*	*y*	*z*	*U*_iso_*/*U*_eq_	
Sn1	1.0000	0.0000	0.0000	0.01123 (3)	
Cl1	0.92766 (3)	0.08742 (2)	0.20722 (2)	0.01631 (6)	
Cl2	1.11712 (3)	0.17373 (2)	−0.06094 (3)	0.01646 (6)	
Cl3	0.78698 (3)	0.06606 (2)	−0.11335 (3)	0.01563 (5)	
N1	0.68736 (10)	0.27997 (8)	0.11589 (10)	0.01418 (17)	
H11	0.7445 (17)	0.2227 (14)	0.1090 (15)	0.025 (4)*	
H12	0.6727 (16)	0.2895 (13)	0.2005 (15)	0.025 (4)*	
C1	0.54865 (11)	0.24787 (9)	0.04603 (11)	0.01420 (19)	
H1	0.5701 (16)	0.2361 (12)	−0.0494 (14)	0.017*	
C2	0.44162 (12)	0.33988 (10)	0.06181 (12)	0.0186 (2)	
H2A	0.4352	0.3571	0.1559	0.025 (2)*	
H2B	0.3517	0.3155	0.0251	0.025 (2)*	
H2C	0.4702	0.4053	0.0144	0.025 (2)*	
C3	0.50358 (13)	0.13875 (10)	0.10779 (13)	0.0213 (2)	
H3A	0.5742	0.0831	0.0957	0.025 (2)*	
H3B	0.4166	0.1148	0.0642	0.025 (2)*	
H3C	0.4914	0.1492	0.2025	0.025 (2)*	
C4	0.75609 (12)	0.38701 (10)	0.07441 (12)	0.0171 (2)	
H4	0.6925 (16)	0.4433 (13)	0.0962 (14)	0.020*	
C5	0.89127 (14)	0.40009 (12)	0.16051 (14)	0.0272 (3)	
H5A	0.8715	0.3948	0.2543	0.036 (3)*	
H5B	0.9321	0.4716	0.1432	0.036 (3)*	
H5C	0.9557	0.3422	0.1384	0.036 (3)*	
C6	0.78081 (14)	0.38572 (12)	−0.07562 (12)	0.0238 (2)	
H6A	0.8376	0.3224	−0.0961	0.035 (3)*	
H6B	0.8281	0.4530	−0.0997	0.035 (3)*	
H6C	0.6924	0.3809	−0.1260	0.035 (3)*	

### Atomic displacement parameters (Å^2^)

**Table:**

	*U*^11^	*U*^22^	*U*^33^	*U*^12^	*U*^13^	*U*^23^
Sn1	0.01016 (5)	0.01241 (5)	0.01133 (5)	0.00038 (3)	0.00261 (3)	0.00032 (3)
Cl1	0.01780 (12)	0.01875 (12)	0.01260 (10)	0.00316 (9)	0.00306 (9)	−0.00157 (9)
Cl2	0.01694 (12)	0.01595 (12)	0.01663 (11)	−0.00356 (9)	0.00218 (9)	0.00126 (9)
Cl3	0.01222 (10)	0.01879 (12)	0.01588 (11)	0.00180 (9)	0.00070 (9)	0.00081 (10)
N1	0.0125 (4)	0.0154 (4)	0.0148 (4)	0.0002 (3)	0.0028 (3)	0.0007 (4)
C1	0.0133 (4)	0.0151 (5)	0.0143 (4)	−0.0012 (4)	0.0026 (4)	−0.0016 (4)
C2	0.0156 (5)	0.0187 (5)	0.0215 (5)	0.0019 (4)	0.0005 (4)	−0.0009 (4)
C3	0.0207 (5)	0.0159 (5)	0.0280 (6)	−0.0038 (4)	0.0079 (5)	0.0012 (5)
C4	0.0151 (5)	0.0135 (5)	0.0231 (5)	−0.0024 (4)	0.0048 (4)	−0.0016 (4)
C5	0.0198 (6)	0.0310 (7)	0.0307 (6)	−0.0091 (5)	0.0012 (5)	−0.0068 (6)
C6	0.0235 (6)	0.0242 (6)	0.0243 (6)	−0.0051 (5)	0.0073 (5)	0.0059 (5)

### Geometric parameters (Å, º)

**Table:**

Sn1—Cl3^i^	2.4055 (3)	C2—H2B	0.9600
Sn1—Cl3	2.4055 (3)	C2—H2C	0.9600
Sn1—Cl1	2.4359 (3)	C3—H3A	0.9600
Sn1—Cl1^i^	2.4359 (3)	C3—H3B	0.9600
Sn1—Cl2	2.4527 (3)	C3—H3C	0.9600
Sn1—Cl2^i^	2.4527 (3)	C4—C6	1.5168 (16)
N1—C4	1.5073 (15)	C4—C5	1.5178 (17)
N1—C1	1.5117 (14)	C4—H4	0.940 (16)
N1—H11	0.881 (16)	C5—H5A	0.9600
N1—H12	0.864 (15)	C5—H5B	0.9600
C1—C3	1.5153 (16)	C5—H5C	0.9600
C1—C2	1.5164 (16)	C6—H6A	0.9600
C1—H1	0.988 (14)	C6—H6B	0.9600
C2—H2A	0.9600	C6—H6C	0.9600
			
Cl3^i^—Sn1—Cl3	180.000 (18)	H2A—C2—H2B	109.5
Cl3^i^—Sn1—Cl1	90.994 (9)	C1—C2—H2C	109.5
Cl3—Sn1—Cl1	89.006 (9)	H2A—C2—H2C	109.5
Cl3^i^—Sn1—Cl1^i^	89.006 (9)	H2B—C2—H2C	109.5
Cl3—Sn1—Cl1^i^	90.994 (9)	C1—C3—H3A	109.5
Cl1—Sn1—Cl1^i^	180.000 (13)	C1—C3—H3B	109.5
Cl3^i^—Sn1—Cl2	90.528 (9)	H3A—C3—H3B	109.5
Cl3—Sn1—Cl2	89.472 (9)	C1—C3—H3C	109.5
Cl1—Sn1—Cl2	89.711 (9)	H3A—C3—H3C	109.5
Cl1^i^—Sn1—Cl2	90.289 (9)	H3B—C3—H3C	109.5
Cl3^i^—Sn1—Cl2^i^	89.472 (9)	N1—C4—C6	110.50 (9)
Cl3—Sn1—Cl2^i^	90.528 (9)	N1—C4—C5	107.69 (10)
Cl1—Sn1—Cl2^i^	90.289 (9)	C6—C4—C5	112.41 (10)
Cl1^i^—Sn1—Cl2^i^	89.711 (9)	N1—C4—H4	104.7 (9)
Cl2—Sn1—Cl2^i^	180.000 (13)	C6—C4—H4	111.5 (9)
C4—N1—C1	118.34 (9)	C5—C4—H4	109.7 (9)
C4—N1—H11	111.2 (10)	C4—C5—H5A	109.5
C1—N1—H11	107.3 (10)	C4—C5—H5B	109.5
C4—N1—H12	104.2 (11)	H5A—C5—H5B	109.5
C1—N1—H12	107.3 (10)	C4—C5—H5C	109.5
H11—N1—H12	108.0 (14)	H5A—C5—H5C	109.5
N1—C1—C3	107.14 (9)	H5B—C5—H5C	109.5
N1—C1—C2	110.28 (9)	C4—C6—H6A	109.5
C3—C1—C2	112.27 (10)	C4—C6—H6B	109.5
N1—C1—H1	104.8 (9)	H6A—C6—H6B	109.5
C3—C1—H1	109.9 (9)	C4—C6—H6C	109.5
C2—C1—H1	112.1 (9)	H6A—C6—H6C	109.5
C1—C2—H2A	109.5	H6B—C6—H6C	109.5
C1—C2—H2B	109.5		
			
C4—N1—C1—C3	−179.42 (9)	C1—N1—C4—C6	−57.67 (13)
C4—N1—C1—C2	−56.96 (12)	C1—N1—C4—C5	179.20 (10)

Symmetry code: (i) −*x*+2, −*y*, −*z*.

### Hydrogen-bond geometry (Å, º)

**Table:**

*D*—H···*A*	*D*—H	H···*A*	*D*···*A*	*D*—H···*A*
N1—H11···Cl1	0.881 (16)	2.541 (16)	3.3449 (10)	152.1 (13)
N1—H12···Cl2^ii^	0.864 (15)	2.488 (15)	3.3507 (10)	176.0 (14)

Symmetry code: (ii) *x*−1/2, −*y*+1/2, *z*+1/2.
